# Total hip replacement in young adults with hip dysplasia: Age at diagnosis, previous treatment, quality of life, and validation of diagnoses reported to the Norwegian Arthroplasty Register between 1987 and 2007

**DOI:** 10.3109/17453674.2011.566146

**Published:** 2011-04-05

**Authors:** Ingvild Ø Engesæter, Trude Lehmann, Lene B Laborie, Stein Atle Lie, Karen Rosendahl, Lars B Engesæter

**Affiliations:** ^1^The Norwegian Arthroplasty Register, Department of Orthopaedic Surgery, Haukeland University Hospital; ^2^Department of Surgical Sciences, University of Bergen; ^3^Department of Radiology, Haukeland University Hospital, Bergen; ^4^Uni Health, Uni Research, Bergen, Norway

## Abstract

**Background and purpose:**

Dysplasia of the hip increases the risk of secondary degenerative change and subsequent total hip replacement. Here we report on age at diagnosis of dysplasia, previous treatment, and quality of life for patients born after 1967 and registered with a total hip replacement due to dysplasia in the Norwegian Arthroplasty Register. We also used the medical records to validate the diagnosis reported by the orthopedic surgeon to the register.

**Methods:**

Subjects born after January 1, 1967 and registered with a primary total hip replacement in the Norwegian Arthroplasty Register during the period 1987–2007 (n = 713) were included in the study. Data on hip symptoms and quality of life (EQ-5D) were collected through questionnaires. Elaborating information was retrieved from the medical records.

**Results:**

540 of 713 patients (76%) (corresponding to 634 hips) returned the questionnaires and consented for additional information to be retrieved from their medical records. Hip dysplasia accounted for 163 of 634 hip replacements (26%), 134 of which were in females (82%). Median age at time of diagnosis was 7.8 (0–39) years: 4.4 years for females and 22 years for males. After reviewing accessible medical records, the diagnosis of hip dysplasia was confirmed in 132 of 150 hips (88%).

**Interpretation:**

One quarter of hip replacements performed in patients aged 40 or younger were due to an underlying hip dysplasia, which, in most cases, was diagnosed during late childhood. The dysplasia diagnosis reported to the register was correct for 88% of the hips.

Hip disorders in children, such as developmental dysplasia of the hip (DDH), Perthes' disease, and slipped capital femoral epiphysis (SCFE) may lead to degenerative changes warranting a total hip replacement (THR) in young adults (Jacobsen et al. 2005). Dysplasia is the more common of these (Thillemann et al. 2008), with a reported prevalence of 0.5–4% according to time and type of ascertainment and the diagnostic criteria used (Rosendahl et al. 1996, Holen et al. 1999, Reikeras et al. 1999, Lehmann et al. 2000). Most patients with dysplasia are diagnosed during the newborn period by clinical screening tests and/or an additional ultrasound examination. However, despite early abduction treatment for those testing positive, the number of children in need of surgical intervention for DDH remains relatively stable, with a reported incidence of around 1 per 1,000 (Godward and Dezateux 1998). As such, the different neonatal screening programs for DDH appear to have failed.

Another issue is whether early abduction treatment may prevent later development of osteoarthritis (OA). OA due to hip dysplasia is reported in up to 8% of adults with a total hip replacement (Furnes et al. 2001, Lohmander et al. 2006, Engesaeter et al. 2008b). Females appear to be at a higher risk of developing OA than males (Furnes et al. 2001, Lohmander et al. 2006). In a previous registry-based study, dysplasia was reported to be the underlying cause in almost one third of all total hip replacements in patients under the age of 60 (Furnes et al. 2001). Studies on quality of life in patients with OA secondary to hip dysplasia (Garbuz et al. 2008, Tellini et al. 2008) have shown that a hip replacement in most cases has a positive effect.

We determined the frequency of DDH as the underlying cause of total hip replacement as reported to the Norwegian Arthroplasty Register for patients aged 40 or younger. Next, we examined the age at DDH diagnosis and the various hip-preserving treatments received before the THR; thirdly, we examined the quality of life for patients with a THR; and finally, we validated the agreement between the dysplasia diagnosis reported by the orthopedic surgeon to the register and the diagnosis stated in the medical records.

## Patients and methods

All patients born after January 1, 1967—which was when the Medical Birth Registry of Norway was established—and registered as having a primary total hip replacement in the Norwegian Arthroplasty Register (NAR) during the period 1987 through 2007, were included in the study. From 2005 to 2007, these individuals were approached by regular mail and invited to participate in a follow-up, including 2 questionnaires: one on the actual hip disease leading to THR and the other on their current general health situation.

### The questionnaires

Data on hip symptoms and clinical history were obtained by self reporting, using both a custom-made questionnaire and a standardized, validated international questionnaire: EQ-5D. The custom-made questionnaire included, among others, a question on whether or not the patient agreed with the diagnosis that was registered in the Norwegian Arthroplasty Register.

The EQ-5D is a descriptive tool assessing 5 dimensions: the level of mobility, self-care, usual activity, pain/discomfort, and anxiety/depression. Each question has 3 levels: no problems, some problems, and severe problems. A single, weighted utility score—the EQ-5D_index_—is calculated from the 5 dimensions (Dolan 1997). The “best imaginable health state” represents individuals who report that they are experiencing the highest level of function for the 5 conditions. The EQ-5D_index_ score for these patients is 100. Death scores zero, but conditions worse than death (< 0) are possible. The results from our study were compared with data from age-matched cohorts (18–39 years) from Sweden (46% women) and the UK (56% women) (Szende and Williams 2004).

### Analysis of questionnaire and collection of data from the medical notes

All patients were asked to confirm the nature of the underlying hip disorder as registered in the NAR. If there was agreement, and in cases with an evident underlying diagnosis such as a femoral neck fracture, rheumatoid arthritis, or ankylosing spondylitis (Mb. Bechterew), collection of additional data from the medical records was judged unnecessary to ensure the quality of the data (n = 155). For the reminder (n = 479), having a diagnosis such as idiopathic osteoarthritis, sequelae of dysplasia with or without dislocation, sequelae of Perthes'/SCFE or “others”, we retrieved additional information from the medical notes in 48 different hospitals throughout the country. Hospitals in which more than 5 patients were operated (n = 14) were visited by one of the authors (IØE, TL). The remaining hospitals (n = 34) were contacted by mail and asked to submit a copy of the medical records. 23 hospitals responded, providing data on 70 of the relevant 91 hips.

The medical records were reviewed by the same 2 authors (IØE, TL). From these records, we retrieved the primary hip diagnosis and classified the diagnosis as either “true”, “false”, or “equivocal”. Diagnoses found in the clinical record, supported by information on clinical findings, symptoms and radiological reports, were accepted as true. In most patients, we were unable to find radiographs taken at the time of diagnosis. This is due to national regulations where radiographs not used in the ensuing 10 years are normally destroyed. Unfortunately, this made it impossible to validate the diagnosis on the basis of the primary radiographs. For patients diagnosed with DDH, Perthes' disease, or SCFE, we also recorded date and symptoms at the time of diagnosis of the hip disease and treatment given for the hip disorder until they underwent THR.

### Diagnoses reported to the Norwegian Arthroplasty Register

Since September 1987, total hip replacement procedures have been reported to the Norwegian Arthroplasty Register (NAR) (Havelin et al. 2000). A standard form including the patient's unique 11-digit identity number, date of surgery, and indication for and type of THR is filled in by the surgeon and submitted to the registry. Both primary operations and reoperations are recorded. Reporting to the NAR is not mandatory, but estimates suggest that there is a reporting frequency of at least 97% of joint replacements performed in Norway (Espehaug et al. 2006). The diagnosis of hip dysplasia is split into 2 categories in the register: dysplasia and dysplasia with dislocation at the time of THR. In our analysis we merged the 2 alternatives, resulting in one output called “DDH”.

### Statistics

Data were summarized using median and range or mean and standard deviation (SD) as appropriate. Comparison of mean values was done using t-tests, where a p-value of < 0.05 indicated a statistically significant difference. The analyses were performed in the statistical program SPSS for Windows version 17.0.

### Ethics

In order to validate data in the Norwegian Arthroplasty Register, the procedures were approved by the Regional Ethical Committee for Medical and Health Research. In addition, all patients returned a written informed consent form where they gave their permission to obtain further information from the medical records of the hospitals. Patients who did not give this consent were excluded from the study (n = 32).

## Results

**Figure 1.**
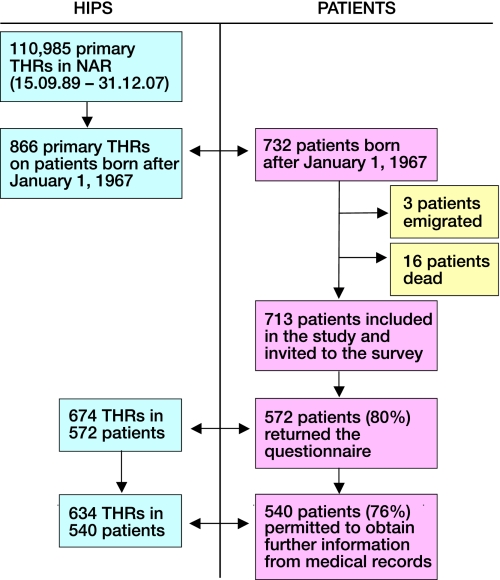
Flow of participants in the study.

732 patients born after January 1, 1967 were registered in the Norwegian Arthroplasty Register as having undergone a primary THR. 540 patients (76%) were included in the study (corresponding to 634 hip replacements) (Figure 1). Mean age was 33 (SD 5.3) years and 300 of these patients were females.

According to the Arthroplasty Register, hip dysplasia accounted for 163 (26%) of the 634 hip replacements (Table 1). We were unable to retrieve extended medical information for 13 hips, leaving us with 150 hips for validation. Of the 150 THRs, the dysplasia diagnosis was confirmed by medical notes in 88% (132 hips, 114 patients). 18 hips (12%) were classified as having incorrect reporting. Of the incorrectly reported hips, reassessment revealed the following diagnoses: Perthes' disease (n = 5), SCFE (n = 1), epiphyseal dysplasia (n = 5), sequelae following previous traumatic dislocation (n = 2), previous avascular necrosis secondary to steroid therapy (n = 3), tuberculous coxitis (n = 1), and myelomeningocele with spasms (n = 1). In addition to those 132 already correctly reported hips, 10 hips were reclassified as hip dysplasia but these were not included in the analyses.

**Table 1.** Diagnosis underlying a total hip replacement in 540 patients, as recorded in the Norwegian Arthroplasty Register (NAR), compared to the diagnoses recorded in the initial medical records. 6 hips were registered with multiple diagnoses

Diagnoses	Diagnosis as registered in NAR (n hips)	Hips available for validation	NAR diagnosis confirmed n (%)	Other diagnosis confirmed n (%)
Idiopathic osteoarthritis	42	31	19 (61)	12 (39)
Rheumatoid arthritis	114	112	109 (97)	3 (3)
Sequelae fractura colli femoris	27	26	25 (96)	1 (4)
Sequelae of dysplasia/sequelae of dysplasia with dislocation	163	150	132 (88)	18 (12)
Sequelae of Perthes'/SCFE	101	94	89 (95)	5 (5)
Ankylosis spondylitis (Mb. Bechterew)	28	28	28 (100)	0 (0)
Acute fractura colli femoris	2	2	2 (100)	0 (0)
Others	160	145	134 (92)	11 (8)
Missing diagnosis	4	4	0 (0)	4 (100)

The accuracy of the other diagnoses registered in the registry varied from 93% to 100% (Table 1), with the exception of the group “idiopathic osteoarthritis” where only 61% of the diagnoses were validated as correct. All the diagnoses in the remaining 39% (12 hips) were judged as error reports. 4 of the incorrectly reported “idiopathic osteoarthritis” hips were validated as sequelae of dysplasia and 8 hips were classified as “other diagnosis”.

**Figure 2.**
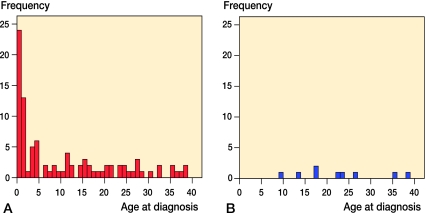
A. Age at diagnosis for females (n = 95). Median age at diagnosis was 4.4 (0–38) years. B. Age at diagnosis for males (n = 9). Median age at diagnosis was 22 (9.5–39) years.

Median age at the time of diagnosis of DDH was 7.8 (0–39) years: 4.4 years for females and 22 for males (Figure 2), with the more common symptoms/findings at presentation being limp (25%), hip pain (20%), and reduced hip abduction (18%).

Data on previous hip-preserving treatment (both surgery and nonoperative treatment) were retrieved for 88 of the 132 confirmed dysplastic hips (67%) (Table 2). Previous surgery was reported for 81 of the 88 hips, femur osteotomy being the most common and seen in 63 (36%) of 176 surgical procedures. (More than 1 procedure during each operation was possible). Rotation and varisation were the two most common subtypes of the femur osteotomies. Different acetabular osteotomies were seen in 24% (43/176 procedures) of which Salter innominate osteotomy dominated (23/176 procedures). 14 hips (19/176 procedures, 11%) had undergone open reduction. In 10 hips (9 patients, 12% of the total) early abduction treatment with a Frejka pillow was given, but all of them were in need of hip preserving surgery later, i.e. none of those reported with only Frejka pillow as treatment received a THR later.

**Table 2.** Treatment prior to THR for the 132 hips with a confirmed DDH in the Norwegian Arthroplasty Register

Hips	n (%)	n (% of 88)
Hip preserving treatment	88 (67)	
Frejka pillow		10 (11)
Hip spica cast		51 (58)
Traction		41 (47)
Orthosis		4 (5)
Hip-preserving surgery		81 (92)
No treatment prior to THR	34 (26)	
No information on prior treatment	10 (8)	
Total	132 (100)	

**Figure 3.**
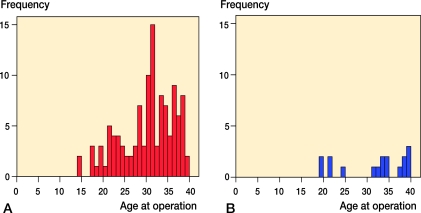
A. A age at THR for females (n=115). Median age of operation 14 (14–40) years. B. Age at THR for males (n=17). Median age of operation 33 (20–39) years.

Median age at the time of THR was 32 (14–40) years; this was similar in both sexes (Figure 3). 18 patients had been operated with bilateral prosthesis.

Regarding the EQ-5D questions, 66% of the patients (n = 75) reported having some pain or severe pain. 67 (59%) had problems with mobility and 62 (54%) had difficulties with daily activities. The mean EQ-5D_index_ for men (score = 74) was slightly better than for women (score = 67), but the difference was not statistically significant (p = 0.2). The self-reported health situation for patients who were operated due to DDH (score = 68) was worse than that reported for the general population aged from 18 to 39 years in Sweden (score = 86, p < 0.001) and in the United Kingdom (score = 87, p < 0.001).

## Discussion

According to data in the Norwegian Arthroplasty Register (NAR), hip dysplasia is the underlying cause in 1 out of every 4 total hip replacements performed in patients aged 40 or younger. The diagnosis registered in the NAR was confirmed in 88% of the cases after review of the medical records. This figure compares favorably with that reported for the Danish Hip Arthroplasty Register, in which the diagnosis was confirmed in 94% of patients with congenital hip dysplasia and in 81% of those with acetabular dysplasia (Pedersen et al. 2004).

The median age at diagnosis of DDH was 7.8 years, which is indeed noteworthy, and in agreement with a previous study using data from the Medical Birth Registry of Norway and the Norwegian Arthroplasty Register (Engesaeter et al. 2008a). That study, involving 442 patients, showed that 92% of all patients who underwent THR due to an underlying dysplasia had no reported hip instability in the newborn period, as assessed on clinical examination. This may be a result of the fact that the clinical screening tests for DDH, i.e. the Barlow/Ortolani maneuvers, have low sensitivity and specificity for DDH (Rosendahl et al. 1996), or it may reflect problems in reporting to or registration in the Medical Birth Registry itself, or a combination of the two. However, the possibility of a stable but dysplastic hip at birth deterioriating throughout childhood, with development of secondary degenerative changes, offers an alternative explanation. Due to missing radiographs in our study, we could not determine whether or not the hip was subluxed or dislocated at the time of diagnosis, or confined to the acetabulum alone, which would obviously have helped in our understanding of the disease.

There are 2 principles of surgical treatment of hip dysplasia: hip preservation surgery and joint replacement. Different techniques with femoral and/or acetabular osteotomies are the most well-established preservation procedures and are optional in severe acetabular dysplasia (Weinstein et al. 2004, Clohisy et al. 2009). Our study revealed that 81 of 132 hips (61%) in young adults had undergone hip preservation surgery before THR was performed. In accordance with the recommended techniques described in the literature, the most common surgical procedures were femoral (rotation and/or varisation) and acetabular osteotomies.

THR is common in patients with DDH, after nonoperative options and/or less invasive surgery have proven to be insufficient. In many respects, our patient group differs from the average patient who has had a THR. They are younger and therefore probably more physically active, and have high demands regarding hip function. This may influence the results of a general health assessment such as the EQ-5D, as our cohort reported relatively low scores for quality of life—of around 67–72 out of 100—as compared to those for the general, age-matched Swedish and UK populations (85–90). Two thirds reported some degree of pain, anxiety, or depression, while three fifths experienced problems with mobility and half had difficulties with daily activities. Such information is crucial for the young DDH patient when different treatment options are being discussed.

The strengths of our study are the population-based design, the high response rate, and the standardized and thorough assessment of an underlying hip diagnosis. In an earlier study, we showed that nearly all total hip replacements are reported to the Norwegian Arthroplasty Register, allowing for generalization of the results (Espehaug et al. 2006). The response rate was high, and there is no reason to believe that the non-responders differed regarding the underlying diagnosis from those who responded. Moreover, the collection of data from previous medical notes as well as reassessment of diagnoses was performed by only 2 physicians/researchers, both with a special interest in DDH.

There were several limitations to our study. First, the dysplasia diagnosis was based on medical records rather than on primary radiographs. During data collection, however, we noticed that most patients with DDH had undergone treatment with follow-up before the THR, which had been well documented in the clinical and radiological notes. We were therefore able to validate the diagnosis without the support of radiographs. Another limitation is that we did not identify whether or not the hip disorder was secondary to a neurological condition, such as cerebral palsy or meningomyelocele. However, these are rare conditions as compared to DDH, with a prevalence of 1–4 per 1,000 term infants (Johnson 2002, Frey and Hauser 2003), and as such would not have influenced our results significantly. Thirdly, missing reports and data are always a concern in cross-sectional studies. We lacked the medical records for 21 hips (3%), 13 of which were registered as dysplastic. These cases were, however, excluded from the study and only patients for whom both questionnaires and medical records existed were included in the final analysis.

In conclusion, we have shown that 1 out of every 4 hip replacements performed in patients aged 40 or younger have been due to an underlying hip dysplasia, and that most of these were diagnosed in late childhood. Three quarters of them had undergone different hip-preserving treatments before their prosthesis. The Arthroplasty Register-based diagnoses were correct in 88% of the cases.
